# Kinetics of the Antibody Response to Boostering With Three Different Vaccines Against SARS-CoV-2

**DOI:** 10.3389/fimmu.2022.811020

**Published:** 2022-01-19

**Authors:** Robert Markewitz, David Juhl, Daniela Pauli, Siegfried Görg, Ralf Junker, Jan Rupp, Sarah Engel, Katja Steinhagen, Victor Herbst, Dorinja Zapf, Christina Krüger, Christian Brockmann, Frank Leypoldt, Justina Dargvainiene, Benjamin Schomburg, Shahpour Sharifzadeh, Lukas Salek Nejad, Klaus-Peter Wandinger, Malte Ziemann

**Affiliations:** ^1^ Institute of Clinical Chemistry, University Hospital of Schleswig-Holstein, Kiel, Germany; ^2^ Institute of Transfusion Medicine, University Hospital of Schleswig-Holstein, Lübeck, Germany; ^3^ Department of Infectious Diseases and Microbiology, University of Lübeck, Lübeck, Germany; ^4^ Department of Anesthesiology and Intensive Care, University Hospital of Schleswig-Holstein, Lübeck, Germany; ^5^ Institute for Experimental Immunology, EUROIMMUN AG, Lübeck, Germany

**Keywords:** SARS-CoV-2, vaccination, B-cell response, immune response, kinetics

## Abstract

**Background:**

Heterologous vaccinations against SARS-CoV-2 with ChAdOx1 nCoV-19 and a second dose of an mRNA-based vaccine have been shown to be more immunogenic than homologous ChAdOx1 nCoV-19. In the current study, we examined the kinetics of the antibody response to the second dose of three different vaccination regimens (homologous ChAdOx1 nCoV-19 vs. ChAdOx1 nCoV-19 + BNT162b2 or mRNA-1273) against SARS-CoV-2 in a longitudinal manner; whether there are differences in latency or amplitude of the early response and which markers are most suitable to detect these responses.

**Methods:**

We performed assays for anti-S1 IgG and IgA, anti-NCP IgG and a surrogate neutralization assay on serum samples collected from 57 participants on the day of the second vaccination as well as the following seven days.

**Results:**

All examined vaccination regimens induced detectable antibody responses within the examined time frame. Both heterologous regimens induced responses earlier and with a higher amplitude than homologous ChAdOx1 nCoV-19. Between the heterologous regimens, amplitudes were somewhat higher for ChAdOx1 nCoV-19 + mRNA-1273. There was no difference in latency between the IgG and IgA responses. Increases in the surrogate neutralization assay were the first changes to be detectable for all regimens and the only significant change seen for homologous ChAdOx1 nCoV-19.

**Discussion:**

Both examined heterologous vaccination regimens are superior in immunogenicity, including the latency of the response, to homologous ChAdOx1 nCoV-19. While the IgA response has a shorter latency than the IgG response after the first dose, no such difference was found after the second dose, implying that both responses are driven by separate plasma cell populations. Early and steep increases in surrogate neutralization levels suggest that this might be a more sensitive marker for antibody responses after vaccination against SARS-CoV-2 than absolute levels of anti-S1 IgG.

## Introduction

Vaccinations against the Severe acute respiratory syndrome coronavirus 2 (SARS−CoV−2) have been approved and administered since the late year 2020 as a promising measure to contain the further spread of the virus as well as to prevent severe cases of coronavirus disease 2019 (COVID-19). Among the first vaccines to be approved were the two mRNA-based vaccines BNT162b2 (Comirnaty; BioNTech/Pfizer, Germany/USA) (1) and mRNA-1273 (Spikevax; Moderna, USA) (2) and the adenoviral vector vaccine ChAdOx1 nCoV-19 (Vaxzevria; Oxford-AstraZeneca, UK/Sweden) (3). Emerging data on efficacy and reactogenicity of these vaccines (4–7) led to changes in official recommendations concerning the administration of these vaccines. Especially reports of cases of vaccine-induced immune thrombotic thrombocytopenia (VITT), mainly in female recipients of ChAdOx1 nCoV-19 below the age of 60 (7), caused the German permanent commission on vaccination (ständige Impfkommission) to recommend that all individuals who had received a first dose of ChAdOx1 nCoV-19 were to receive a second dose of either BNT162b2 or mRNA-1273, while the use of ChAdOx1 nCoV-19 is generally recommended for recipients ≥ 60 years only.

Studies examining the effect of the different vaccination regimens that resulted from these recommendations (homologous ChAdOx1 nCoV-19 or ChAdOx1 nCoV-19 plus either BNT162b2 or mRNA-1273) have unanimously found heterologous vaccination with ChAdOx1 nCoV-19 and an mRNA-based vaccine induces greater immune responses than homologous ChAdOx1 nCoV-19 (8–11). For the comparison between heterologous regimens, there is still a scarcity of data. However, we were able to find in a previous study (manuscript currently under review) that ChAdOx1 nCoV-19 plus mRNA-1273 induces slightly higher levels of antibodies against SARS-CoV-2 than the respective combination with BNT162b2. As many of these studies have been conducted cross-sectionally, examining only one or two time points since the second vaccination, questions remain whether these differences are caused by different latencies of the responses to the different vaccines or whether they are independent of the time point of sample collection.

In the current study, we examined the intraindividual kinetics of the antibody response to different doses of the second vaccination against SARS-CoV-2. We collected and examined serum samples over the period of 8 days, starting on the day of the second dose, from recipients of anti-SARS-CoV-2 vaccines. As a study collective, individuals who had received ChAdOx1 nCoV-19 as a first dose (and could therefore be expected to exhibit comparable baseline values at the time of the second dose) and were due to receive either ChAdOx1 nCoV-19, BNT162b2, or mRNA-1273 as a second dose were chosen.

With the collected data, we addressed the following questions:
-Do all vaccination regimens induce detectable antibody responses in the examined period of time?
-Are there differences in the kinetics of the antibody response between recipients of different vaccines as second dose? And if yes, are they differences in latency or amplitude of the response?
-What markers are suitable to detect an early response to the second dose and at what point in time can a response be expected?
-Does the recipients’ sex or age influence the kinetics of the antibody response?

## Methods

### Study Population

**Figure 1:**
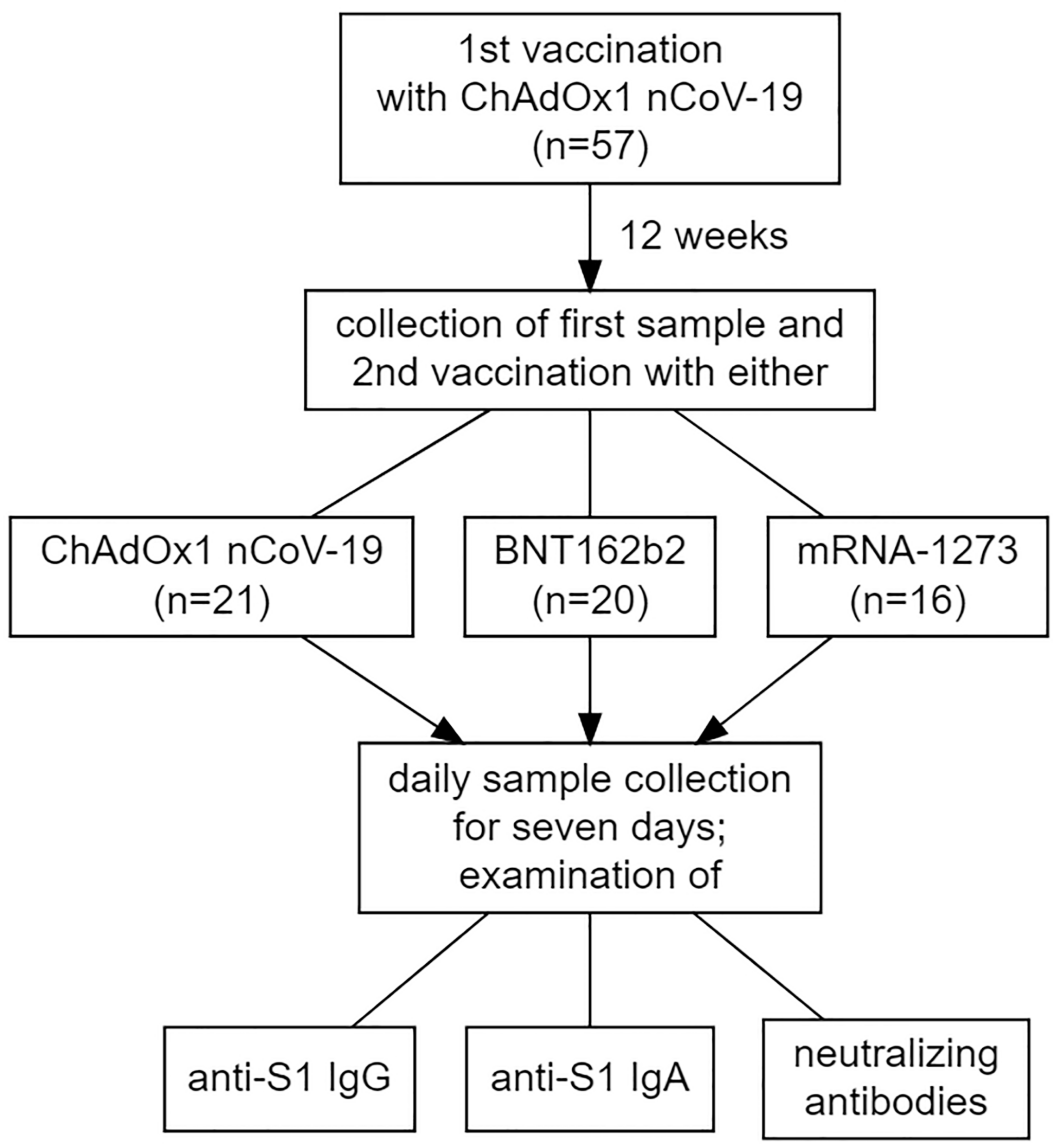
Visualization of the study design, including the makeup of the study cohort concerning the vaccination regimens administered.

Participants were recruited from health care professionals working at the University Hospital Schleswig-Holstein (Lübeck, Germany) who received a second dose of a vaccine against SARS-CoV-2 after having received a first dose of ChAdOx1 nCoV-19. The interval between the two doses were twelve weeks for these vaccinees (see **Figure 1**). Despite the official recommendation that these individuals should receive either BNT162b2 or mRNA-1273 as a second dose, recipients were also free to make an informed decision to receive a second dose of ChAdOx1 nCoV-19 (as only this regimen was approved by the European Medical Agency). Participants who accepted the offer to receive a second dose of an mRNA-based vaccine were not able to choose between BNT162b2 or mRNA-1273.

Prior to participation, all participants gave written informed consent to all procedures they underwent. The study was approved by the University of Kiel institutional review board (AZ: D499/20) and performed in accordance with the declaration of Helsinki (12).

### Sample Characteristics

Serum samples were collected at eight different time points: on the day of the second vaccination (but immediately before it) and on each of the following seven days. Subsequent to collection, the samples were pseudonymized, centrifuged and stored at 4°C until assays were performed. All reported assays (anti-S1 IgA and IgG, anti-NCP IgG and the surrogate neutralization assay) were performed from serum.

### Anti-SARS-CoV-2 Antibodies

Antibodies of the classes IgA and IgG against the S1 subunit of the Spike protein of SARS-CoV-2 (anti-S1) as well as IgG against the nucleocapsid (anti-NCP) antigen were measured from the serum samples using the Anti-SARS-CoV-2-ELISA IgA and Anti-SARS-CoV-2-QuantiVac-ELISA (IgG) test kits by EUROIMMUN (Lübeck, Germany) according to the manufacturers’ instructions. Anti-S1 IgA was performed as a possible early marker of the B-cell response to exposure to SARS-CoV-2 specific antigens. Anti-NCP IgG was performed to exclude any past exposure to the virus itself. More information on the dimension of the reported results as well as their interpretation can be found in the supplement.

### Surrogate Neutralization Assay

The capacity of the anti-S1 antibodies to potentially neutralize SARS-CoV-2 was tested *via* a surrogate neutralization assay (NeutraLISA, EUROIMMUN, Lübeck, Germany). A more detailed description of this assay is contained in the supplement. This test yields a quantitative result reported as a calculated percentage of antibody-induced neutralization. According to the manufacturer, there is a concordance of 98.6% between this method and the examination of neutralizing antibodies *via* plaque reduction neutralization test (PRNT50) (13). The choice to perform a surrogate neutralization assay rather than a neutralization assay was motivated both by reasons of practicability and by the fact that, due to its very limited availability, assays like the PRNT are not likely to be included in routine examinations of vaccine response, which might be different for surrogate neutralization assays which are much easier to implement on a larger scale.

### Statistical Analysis

To analyze the influence of one or more factors on continuous variables, analyses of variance (ANOVAs) were calculated. If the influence of more than one factor was examined, the resulting p-values were adjusted for multiple comparisons using the method described by Benjamini and Yekutieli (14). If exploratory analyses revealed significant main or interaction effects, *post-hoc* testing *via* Tukey’s honest significant differences, a single-step statistical test adjusting for multiple comparisons, was applied. To analyze differences in the distribution of categorically scaled variables between groups, Pearson’s Chi-squared test was used. To analyze the association between two continuous variables, correlations using Spearman’s rho were calculated. For the interpretation of Spearman’s rho, the rule of thumb suggested by Rea and Parker (15) was used (0.0 < 0.1: negligible; 0.1 < 0.2: weak; 0.2 < 0.4: moderate; 0.4 < 0.6: relatively strong; 0.6 < 0.8: strong; 0.8 < 1.0: very strong). Statistical significance was assumed for p-values <0.05. Average values with corresponding measures of dispersion are reported as medians with the median absolute deviation (MAD), unless otherwise stated. All statistical analyses were performed using the open-source software for statistical computing and graphics R (version 4.1.0) with the integrated development environment RStudio (Version 1.4.1717) (16).

## Results

### Study Population

For the current study, 57 participants were included, of which 34 (59.6%) were female. Their median age was 40 years old ( ± 17.8; range: 21-63 years old). Of the 57, 21 (36.8%) received ChAdOx1 nCoV-19 as second vaccine, 20 (35.1%) received BNT162b2 and 16 (28.1%) received mRNA-1273 (See **Table 1** for the numerical makeup of the study cohort, including median ages). A two-way ANOVA with the factors sex and type of second vaccine revealed no significant main or interaction effects of either of these factors on participants’ age (i.e. there was no significant difference in age between recipients of the different vaccination regimens as well as between the two sexes). Pearson’s chi-squared test revealed a slight imbalance in the distribution of sexes between the different vaccination regimens (chi-squared = 6.5101, df = 2, p = 0.03858). This is due to only 38.1% of recipients of ChAdOx1 nCoV-19 as second dose being female, compared to 70% for BNT162b2 and 75% for mRNA-1273. None of the participants showed an anti-NCP IgG response at any time point, suggesting that no participant was exposed to SARS-CoV-2, neither prior to or during the study. Information on missing data can be found in the supplement.

**Table 1:** Overview of the number and respective median ages (including the median absolute deviation as a measure of dispersion), both of the whole cohort and each individual subgroup (recipients of the different vaccination regimens and members of the two sexes).

	Second vaccine:			Whole cohort
	ChAdOx1 nCoV-19	BNT162b2	mRNA-1273	
n (total)	21	20	16	57
n (female)	8	14	12	34
n (male)	13	6	4	23
Median age (total)	47 ± 20	38.5 ± 8.9	39 ± 10.4	40 ± 17.8
Median age (female)	47 ± 17.8	40 ± 10.4	41 ± 13.3	42 ± 13.3
Median age (male)	53 ± 13.3	33 ± 10.4	32 ± 14.8	40 ± 23.7

### Kinetics of the Antibody Response Depending on the Vaccination Regimen

**Figure 2:**
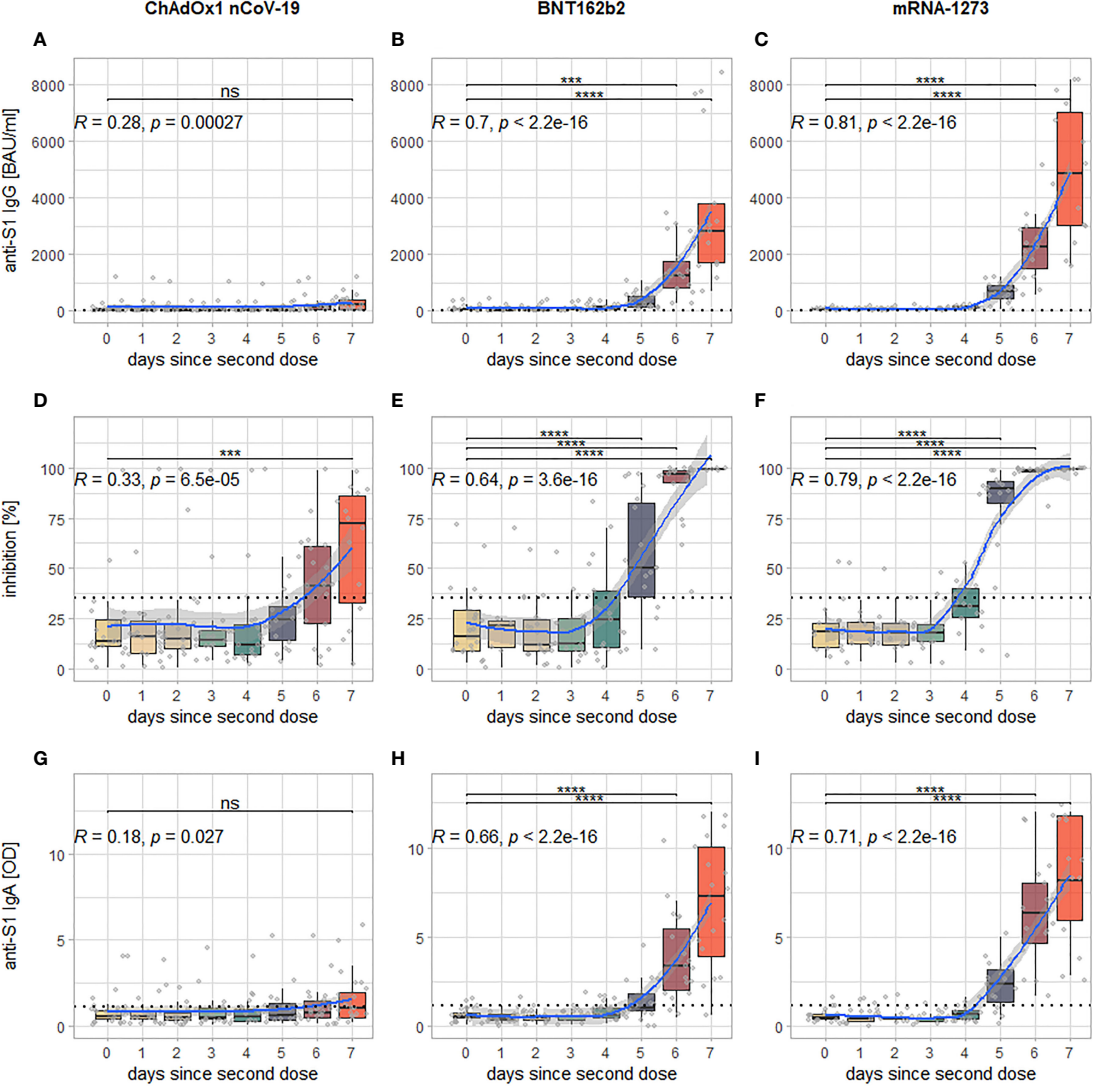
Visualization of the kinetics of all examined markers for the different vaccination regimens: each column of panels represents data from participants who have received either ChAdOx1 nCoV-19 **(A, D, G)**, BNT162b **(B, E, H)** or mRNA-1273 **(C, F, I)** as second dose; each row represents one examined marker: anti-S1 IgG **(A–C)**, inhibition *via* surrogate neutralization assay **(D–F)**, and anti-S1 IgA **(G–I)**. Within each panel, every boxplot represents one time point of sample collection, the individual results are additionally plotted as grey dots. The blue line indicates the smoothed means with a 95% confidence band in light grey. The dotted horizontal line indicates the cutoff for positivity for each assay. In the upper left-hand corner is Spearman’s rho of the correlation between measured levels of the examined marker and days since second dose (along with the associated p-value), the brackets above the boxplots indicate which comparisons between individual time points reveal significant differences (corrected for multiple comparisons). Levels of significance: ****p < 0.0001; ***p < 0.001; ns, not statistically significant.

Three-way ANOVAs with the factors second vaccine, days since second dose and sex revealed statistically highly significant main effects for the factors second vaccine and days since second dose, as well as a significant interaction effect between these two factors for all examined markers (p (adj.) for all comparisons < 0.0001). *Post-hoc* testing (Tukey’s honest significant differences) showed that this was due to the levels of all markers rising significantly in the course of the seven days post second dose. Further exploring the data, it can be seen, however, that this significant rise happens only after second vaccination with BNT162b2 or mRNA-1273, for which a significant increase in observed levels can be detected after six days (anti-S1 IgG and IgA) or five days (neutralizing antibodies) after the second dose with highly significant correlations of strong to very strong effect size between days since second dose and the respective marker (See **Figures 2B, C, E, F, H, I**). For second vaccination with ChAdOx1 nCoV-19, a significant increase in levels from day 0 to day 7 post second dose could only be shown for neutralizing antibodies and correlations between days since second dose and the examined markers, while statistically significant, were only weak to moderate (See **Figures 2A, D, G**).

**Figure 3:**
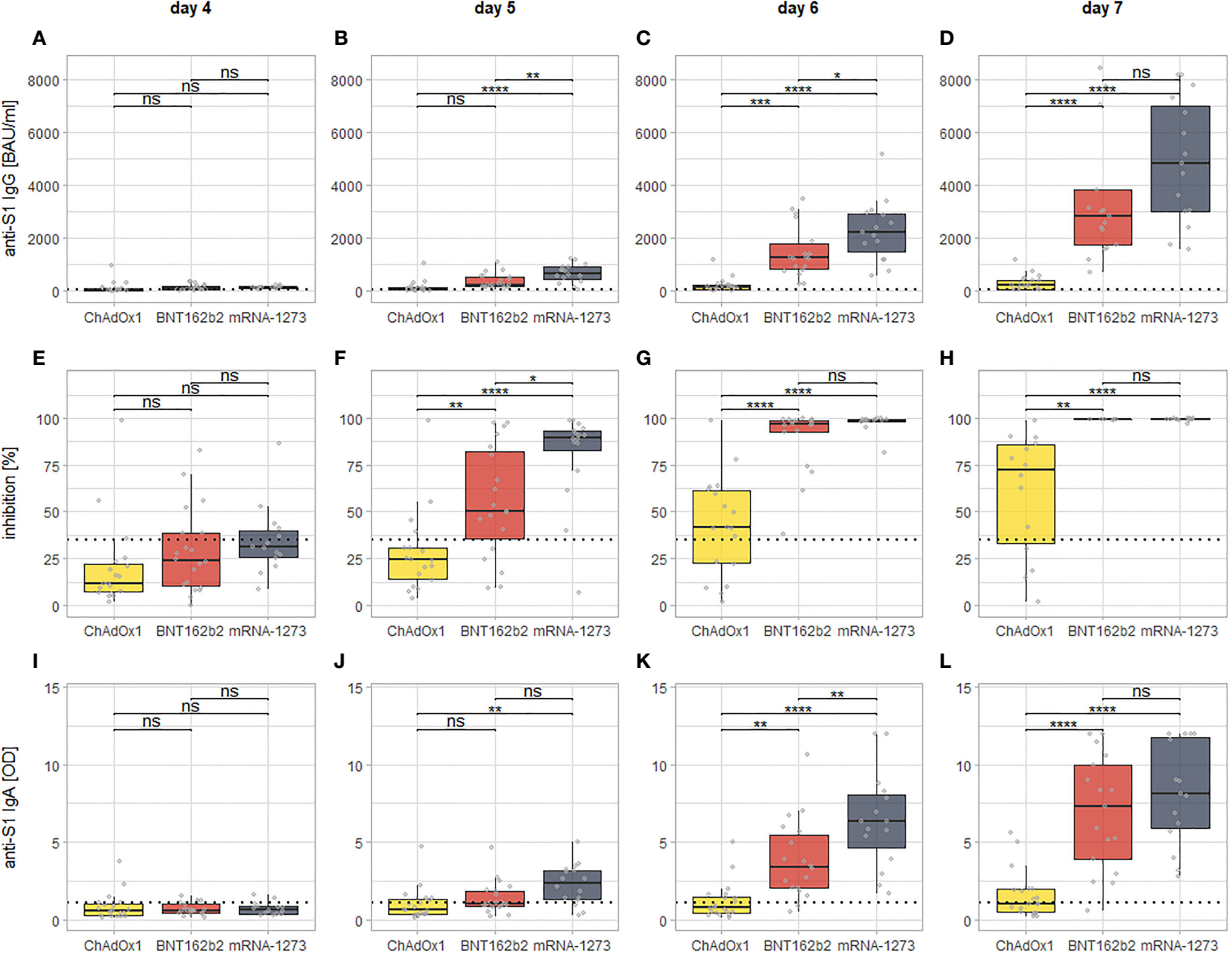
Visualization of the day by day comparison of all examined markers for all of the three vaccination regimens from day four since the second dose onward. Each column of panels represents results for a single time point since the second dose (day 4: **A, E, I**; day 5: **B, F, J**; day 6: **C, G, K**; day 7: **D, H, L**), while each row represent results for a specific assay (anti-S1 IgG: **A–D**; inhibition *via* surrogate neutralization assay: **E–H**; anti-S1 IgA: **I–L**). The dotted lines indicate the cutoffs for positivity for each assay. The brackets indicate the results of *post-hoc* testing for statistically significant differences (via Tukey’s Honest significant differences). Levels of significance: ****p < 0.0001; ***p < 0.001; **p < 0.01; *p < 0.05; ns, not statistically significant.

The *post-hoc* testing further showed that there were no statistically significant differences between recipients of the different second vaccines in any of the observed markers until (and including) day four post second dose. From day five onward, recipients of mRNA-1273 as second dose develop significantly higher levels of all examined markers compared to recipients of ChAdOx1 nCoV-19. For BNT162b2, the same is true at day six (anti-S1 IgG and IgA) or day five (neutralizing antibodies), respectively (See **Figure 3**). The comparison between both mRNA-based vaccines mRNA-1273 and BNT162b2 shows that the former intermittently induces higher levels of all examined markers at day five (neutralizing antibodies), day six (anti-S1 IgA), or both day five and six (anti-S1 IgG), while at day seven after the second dose, there is no statistically significant difference anymore (although a visual trend in favor of mRNA-1273 is still discernible, see **Figure 3**). Of note, a visualization of the same comparisons for days 0-3 can be found in **Figure S1** of the supplement.

### Influence of Sex on the Immune Response

The aforementioned three-way ANOVAs with the factors second vaccine, days since second dose and sex reveals a significant main effect of sex (F = 9.332, df = 1, p (adj.) = 0.006) only on neutralizing antibodies. *Post-hoc* testing shows that this significant main effect of sex is due to men exhibiting slightly higher levels neutralizing antibodies than women (25.2 ± 22.5% vs. 20.9 ± 20.7%), if values are viewed across all time points and vaccination regimens. However, this difference was not found either for one of the three vaccination regimens or one of the eight time points separately.

For anti-S1 IgG and IgA, there was no significant main or interaction effect of sex.

### Influence of Age on the Immune Response

Due to the strong influences of the day since second dose and the vaccine administered as second dose, a possible influence of age on the kinetics of the antibody response to the second vaccination against SARS-CoV-2 was difficult to analyze. Correlations of all examined markers across all vaccination regimens calculated for each day since the second dose revealed significant negative correlations of weak to moderate effect size only between age and anti-S1 IgG on day six (R = -0.29, p = 0.0329) as well as between age and anti-S1 IgA on days six (R = -0.33, p = 0.0165) and seven (R = -0.32, p = 0.019).

## Discussion

Our results show that all of the examined vaccination regimens elicit a detectable antibody response within seven days after the administration of the second dose. Further inspection, however, reveals significant differences in between the three examined vaccination regimens: For the mRNA-based vaccines mRNA-1273 and BNT162b2, significant increases in all examined markers can already be seen at day six after the second dose (or even day five for neutralizing antibodies), only a very weak increase can be detected for anti-S1 IgG and IgA seven days after the second dose of ChAdOx1 nCoV-19. There is, however, a significant increase in levels of neutralizing antibodies at day seven for ChAdOx1 nCoV-19.

Therefore, the differences between the second doses of mRNA-1273 and BNT162b2 that can be seen at day five and six (and that continue to be detectable at 14 days after the second dose (manuscript currently under revision) are a matter of the amplitude of the antibody response (with a stronger response for mRNA-1273), and not its respective latency. The difference between ChAdOx1 nCoV-19 and either of the mRNA-based vaccines (but especially mRNA-1273) is both in latency and in amplitude of the measured responses. One explanation for this difference might be found in the possibility of immune responses against the adenoviral vector impairing the induction of the desired immune response to the vaccine. While the use of a chimpanzee adenoviral vector all but precludes the possibility of preexisting immunity against the vector of ChAdOx (17), the reutilization of the same vector for the second dose might give rise to antivector immunity that interferes with vaccine delivery (18). Other manufacturers have employed different adenoviral vectors for prime and boost doses to circumvent this phenomenon (19, 20). A possible implication is that a second vaccination with an mRNA-based vaccine provides clinical protection earlier than ChAdOx1 nCoV-19, as it has been shown that clinical protection correlates well with the measured levels of anti-S1 IgG (21). Whether or not the observed differences between both mRNA-based vaccines are clinically relevant remains debatable.

There are some surprising findings in the data: While we found in an earlier study that anti-S1 IgA responses after a first dose of an mRNA based vaccine precedes the anti-S1 IgG response (22), we could not find any difference in latency between the IgG and the IgA response in the current study. A possible explanation is the recent finding that a first dose of BNT162b2 induces an IgA-dominant plasmablast response (mainly against the S2 epitope), which might represent a recall response of mucosal memory B-cells formed in response to previous pulmonary coronavirus infections, whereas the (neutralizing) anti-S1 response (IgA and IgG) most likely stems from naïve B-cells which are recruited after the first dose and boostered after the second (23). The role of IgA in respiratory infections is not completely understood, but it is assumed that it acts as a first line of defense on muscosal tissues (24). More research is certainly warranted on the effects of vaccine-induced anti-S1 IgA more, as the focus of research to date was mainly on IgG.

Another intriguing finding is that the ability of the induced antibodies to inhibit binding between S1 and ACE2 *in vitro* during the surrogate neutralization assay increases earlier and more strongly than the overall antibody response (anti-S1 IgG and IgA). For ChAdOx1 nCoV-19 this is even the only response for which significant increases can be shown, with only weak increases of anti-S1 IgG or IgA. This suggests that the second exposure to the S1-antigen *via* the second vaccination preferably induces the production of antibodies with a high affinity to the S1-antigen of which smaller quantities are needed to inhibit the binding between S1 and ACE2. It is possible, therefore, that apart from the quantitative IgG response, as measured *via* international binding antibody units per milliliter, the qualitative ability to inhibit the binding of the virus might be a more sensitive marker of the immune response after vaccination, especially as quantitative levels of IgG wane over time. This assumption is supported by the finding that results of neutralization assay permit a good prediction of protective immunity against SARS-CoV-2 (25, 26). It is important to note however, that to date there are no reliable thresholds for either anti-S1 IgG or neutralizing antibodies (via surrogate neutralization assay) above which a certain degree of clinical protection can be assumed.

Our study has several limitations: Due to considerations of practicability, we did not examine any part of the T-cell response after the second vaccination. Data we gathered for the first dose of the vaccine suggest that the T-cell response might be detectable even earlier after the second dose than the examined antibody response (22). We did not perform a neutralization assay in the proper sense, but rather a surrogate neutralization assay. However, as mentioned, according to the manufacturer, there is a very good concordance between the assay we used and PRNT, one of the methods of choice for neutralization assay. Further, our sample size was quite small, therefore it is possible that we overlooked smaller differences between certain groups. Whether such differences are of clinical significance might be debated however. Lastly, both due to the sample size and because of the fact that the oldest participant was 63 years of age, possible effects of old age might have been overlooked in this study.

In conclusion, we were able to show that all three examined vaccination regimens are able to induce a significant antibody response within a short period of time after the second dose. In between the different vaccination regimens, there are significant differences in latency and amplitude of the response (for the comparison between ChAdOx1 nCoV-19 and both mRNA-based vaccines) or mainly of the amplitude of the response (for the comparison between mRNA-1273 and BNT162b2). Whether these differences are of clinical significance for the protection against SARS-CoV-2 is unclear, however. Lastly, our data suggest that surrogate neutralization assays like the one we used might be used as an early and sensitive marker of the antibody response to the second dose of the vaccination against SARS-CoV-2.

## Data Availability Statement

The raw data supporting the conclusions of this article will be made available by the authors, without undue reservation.

## Ethics Statement

The studies involving human participants were reviewed and approved by University of Kiel institutional review board. The patients/participants provided their written informed consent to participate in this study.

## Author Contributions

RM, MZ, DJ, DP, SS, K-PW, BS, FL, and RJ designed the study. RM, MZ, DJ, CB, JD, SE, JR, and SG contributed to recruitment of participants. The described vaccinations were performed under the direction of SG, MZ, and DJ. The assays were performed by RM, MZ, DJ, DP, KS, VH, DZ, and CK. Data analysis was performed by RM, MZ, DJ, KS, VH, and DZ. The manuscript was written by RM, with support from all authors. All authors have read and approved the final version of the manuscript.

## Conflict of Interest

KS, VH, DZ, and CK currently are employees of the EUROIMMUN AG (Lübeck, Germany).

The remaining authors declare that the research was conducted in the absence of any commercial or financial relationships that could be construed as a potential conflict of interest.

## Publisher’s Note

All claims expressed in this article are solely those of the authors and do not necessarily represent those of their affiliated organizations, or those of the publisher, the editors and the reviewers. Any product that may be evaluated in this article, or claim that may be made by its manufacturer, is not guaranteed or endorsed by the publisher.
