# Supplementary material for: Kinetics of the Antibody Response to Boostering With Three Different Vaccines Against SARS-CoV-2
Source: Front Immunol. 2022 Jan 19;13:811020. doi: 10.3389/fimmu.2022.811020 (PMC8807650; doi:10.3389/fimmu.2022.811020)
Supplement: Supplementary file 1 [file DataSheet_1.docx]

**Supplementary Appendix to:**

**“Kinetics of the antibody response to boostering with three different vaccines against SARS-CoV-2”**

**Table of contents:**

[Methods: 2](#_Toc84238062)

[*Anti-SARS-CoV-2 antibodies* 2](#_Toc84238063)

[*Surrogate neutralization assay* 2](#_Toc84238064)

[Results: 2](#_Toc84238065)

[*Missing data* 2](#_Toc84238066)

[Supplemental Figures: 3](#_Toc84238067)

# Methods:

## *Anti-SARS-CoV-2 antibodies*

The semiquantitative results of both the anti-S1 IgA assay, and the anti-NCP IgG assay are reported as the optical density (OD) of the sample compared to that of a control. The quantitative results of the anti-S1 IgG assay are reported in binding antibody units per milliliter (BAU/ml), ensuring international comparability of the results. For anti-S1 IgA and anti-NCP IgG, values of >1.1 OD are considered as reactive, values of <0.8 OD as negative and all values in between as borderline. For anti-S1 IgG, values of >35.2 BAU/ml are considered as reactive, values of <25.6 BAU/ml as negative and all values in between as borderline.

## *Surrogate neutralization assay*

The surrogate neutralization assay used during the current study (NeutraLISA, EUROIMMUN, Lübeck, Germany) is a competitive ELISA: A buffer containing soluble biotinylated ACE2 is added to the serum sample that is to be examined. Subsequently, the mixture of sample and buffer is incubated in wells coated with the recombinant S1 subunit of the Spike protein of SARS-CoV-2, where the ACE2 in the buffer competes with anti-S1 IgG in the sample for the binding at the recombinant that is attached to a solid phase. After a washing step, the amount of ACE2 that has bound to the recombinant S1 of the solid phase and is therefore is still detectable via an enzymatic reaction, using streptavidin-coupled peroxidase, is inversely proportional to the concentration of neutralizing antibodies. The signal detected in samples is compared to the mean signal of a duplicate measurement of the buffer alone (blank), for which the highest possible signal is expected. The result is reported as inhibition [%], which is calculated via the following formula: $\mathrm{inhibition}\left[ \% \right]= 100 \%- \frac{extinction of the sample \times100 \%}{mean extinction of the blank}$. Values < 20 % are considered non-reactive, values ≥ 35 are considered reactive and values between 20 and 35 % are considered to be borderline.

# Results:

## *Missing data*

Not all 57 participants were able to donate samples at all of the eight time points. While samples from all participants were available at baseline (day 0), samples from 53 (93 %) participants were available at day 1, from 55 (96.5 %) at day two, from 54 (94.7 %) at days three and four, from 56 (98.2 %) at day five, from 53 (93 %) at day six and from 52 (91.2 %) participants at day seven after the second dose. Of the 57 participants, 44 (77.2 %) completed all eight time points, nine (15.8 %) completed seven time points, one (1.8 %) completed six time points, two (3.5 %) completed five time points and one (1.8 %) completed three time points. In handling missing data, an available case analysis (also known as pairwise deletion) was applied, i.e. cases are included for analyses if all required variables are present or not included of one variable is missing.

# Supplemental Figures:


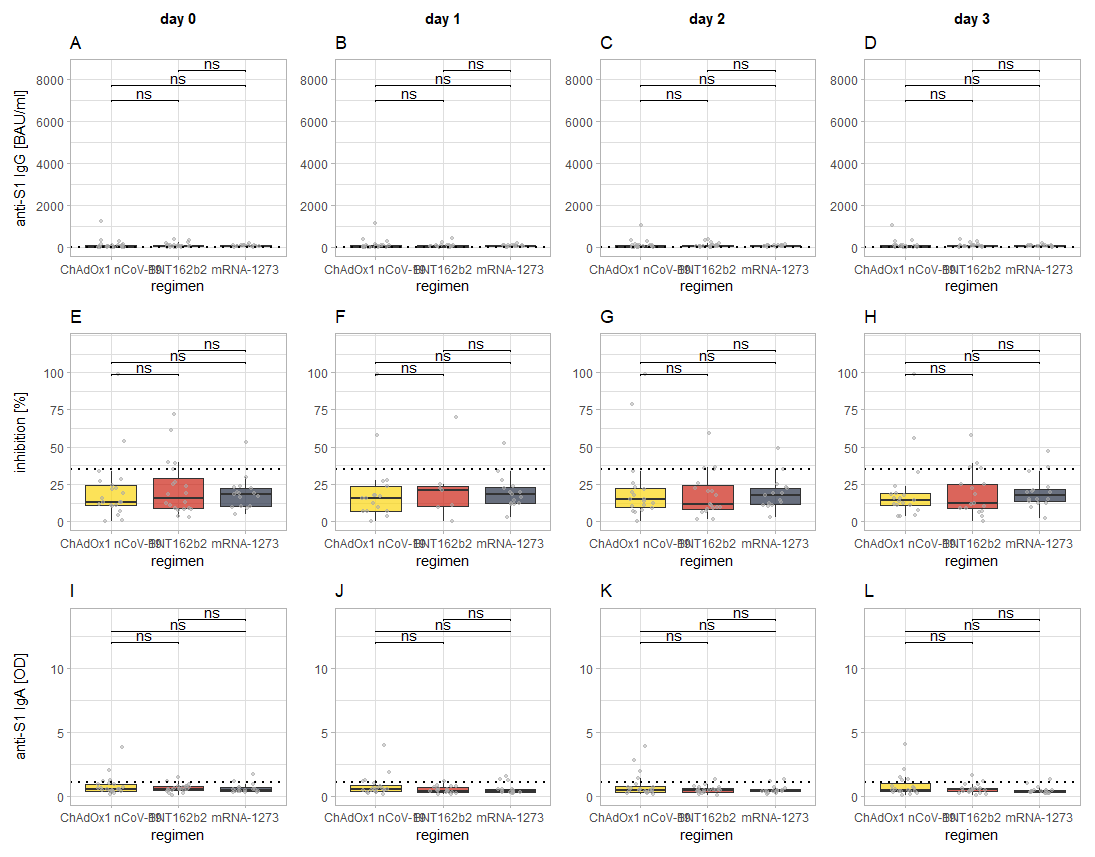


**Fig S1:** visualization of the day by day comparison of all examined markers for all of the three vaccination regimens from day 0 to day 3 since the second dose. Each column of panels represents results for a single time point since the second dose (day 4: panels A, E, and I; day 5: panels B, F, and J; day 6: panels C, G, and K; day 7: panels D, H, and L), while each row represent results for a specific assay (anti-S1 IgG: panels A-D; inhibition via surrogate neutralization assay: panels E-H; anti-S1 IgA: panels I-L). The dotted lines indicate the cutoffs for positivity for each assay. The brackets indicate the results of post-hoc testing for statistically significant differences (via Tukey’s Honest significant differences). Levels of significance: ns = not statistically significant.
